# Genome-wide identification and expression analysis of peach multiple organellar RNA editing factors reveals the roles of RNA editing in plant immunity

**DOI:** 10.1186/s12870-022-03982-2

**Published:** 2022-12-13

**Authors:** Aidi Zhang, Yuhong Xiong, Jing Fang, Kangchen Liu, Huixiang Peng, Xiujun Zhang

**Affiliations:** 1 Key Laboratory of Plant Germplasm Enhancement and Specialty Agriculture, Wuhan Botanical Garden, Chinese Academy of Sciences, Wuhan, 430074 China; 2 Center of Economic Botany, Core Botanical Gardens, Chinese Academy of Sciences, Wuhan, 430074 China; 3 University of Chinese Academy of Sciences, Beijing, 100049 China

**Keywords:** Peach, Multiple organellar RNA editing factor, RNA editing, Pathogens stress, Plant immunity

## Abstract

**Background:**

*Multiple organellar RNA editing factor* (*MORF*) genes play key roles in chloroplast developmental processes by mediating RNA editing of Cytosine-to-Uracil conversion. However, the function of *MORF* genes in peach (*Prunus persica*), a perennial horticultural crop species of Rosaceae, is still not well known, particularly the resistance to biotic and abiotic stresses that threaten peach yield seriously.

**Results:**

In this study, to reveal the regulatory roles of RNA editing in plant immunity, we implemented genome-wide analysis of peach *MORF (PpMORF)* genes in response to biotic and abiotic stresses. The chromosomal and subcellular location analysis showed that the identified seven *PpMORF* genes distributed on three peach chromosomes were mainly localized in the mitochondria and chloroplast. All the *PpMORF* genes were classified into six groups and one pair of *PpMORF* genes was tandemly duplicated. Based on the meta-analysis of two types of public RNA-seq data under different treatments (biotic and abiotic stresses), we observed down-regulated expression of *PpMORF* genes and reduced chloroplast RNA editing, especially the different response of *PpMORF2* and *PpMORF9* to pathogens infection between resistant and susceptible peach varieties, indicating the roles of *MORF* genes in stress response by modulating the RNA editing extent in plant immunity. Three upstream transcription factors (*MYB3R-1*, *ZAT10*, *HSFB3*) were identified under both stresses, they may regulate resistance adaption by modulating the *PpMORF* gene expression.

**Conclusion:**

These results provided the foundation for further analyses of the functions of *MORF* genes, in particular the roles of RNA editing in plant immunity. In addition, our findings will be conducive to clarifying the resistance mechanisms in peaches and open up avenues for breeding new cultivars with high resistance.

**Supplementary Information:**

The online version contains supplementary material available at 10.1186/s12870-022-03982-2.

## Background

Peach (*Prunus persica*) is a deciduous tree or shrub in the rose family grown for its edible fruit with high minerals, vitamins, fiber, and antioxidant compounds, and it is native to China where is also the world’s largest producer and consuming country. Peach is usually used as the model species in Rosaceae with special characteristics such as self-pollinate ability, a short life cycle, and a small genome size of 265 Mb [1, 2]. However, peach is often subject to various stresses during the growing season, such as pathogens infection, light intensity, low or high temperature, and so on, limiting the growth and yield of peach. Being one of the biotic stresses, bacterial perforation disease caused by *Xanthomonas arboricola pv. Pruni* (*Xap*) is one of the most serious diseases in peach, which often causes leaf perforation, affects the normal growth of fruit or flower bud differentiation and development, and leads to flower drop and fruit quality deterioration, resulting in substantial economic losses worldwide [3]. Being one of abiotic stresses, ultraviolet radiation affect the growth of most plants including peach, which showed decreased plant height, leaf area, photosynthetic rate, and productivity when exposed to ultraviolet radiation [4]. These stresses are destructive and economically damaging for peaches. However, the resistance mechanism in response to multiple stresses remains unclear.

RNA editing is a type of post-transcriptional modification which is mainly manifested as nucleotide insertion/deletion or conversion, yielding genetic information on RNA products that are different from their DNA templates [5]. In flowering plants, the post-transcriptional modification includes C-to-U (Cytosine-to-Uracil), U-to-C (Uracil-to- Cytosine), and A-to-I (Adenosine-to-Inosine) editing, and there are about 400–500, 30–40 C-to-U editing in transcripts of mitochondria and chloroplast respectively [6–8]. RNA editing plays an indispensable role in plant organelle biogenesis, adaptation to environmental changes, and signal transduction. Many mutants with impaired editing of specific sites exhibited strong deleterious phenotypes, even lethality [9–12]. In our previous studies, we found that the RNA editing events in grapes were reduced in response to heat stress [11], the RNA editing events in kiwifruits were reduced in response to pathogens infection [13].

In flowering plants, RNA editing is mainly mediated by editing complexes involving multiple editing factors, including pentatricopeptide repeat (PPR), organelle zinc finger (OZ), organelle RNA recognition motif-containing protein (ORRM), protoporphyrinogen IX oxidase (PPO), and multiple organellar RNA editing factors (MORF) [9, 14–17]. A PPR protein utilizes its DYW domain to recognize the five cis-elements upstream of the edited cytosine, which is essential in the post-transcriptional regulation of mitochondrial and chloroplast RNA [18]. MORF protein binds to the DYW domain of PPR protein to modulate the RNA-binding activity. The loss of a MORF protein will abolish or lower editing at multiple sites, previous studies found that disruption of *MORF8*, *MORF3* and *MORF1* genes reduced 72%, 26% and 19% of mitochondria editing events respectively, whereas mutants of either *MORF2* or *MORF9* exhibited reduced editing at almost all sites in chloroplasts [8, 14].

The *MORF* gene family has been widely identified in plants, such as *Arabidopsis* with 9 members, *Populus trichocarpa* with 9 [9], *O. sativa* with 7 [19], *Z. mays* with 7 [20], *Actinidia chinensis* with 10 [13] and *Nicotiana* with 8 [21]. The crystal structures of MORF1/MORF9 protein complex in *Arabidopsis* were determined [17], which showed that they both adopt a novel globular fold, and validated the mechanism of MORF proteins multimerization. In *Arabidopsis*, *MORF2* and *MORF9* are targeted to the chloroplast, *MORF5* and *MORF8* are localized in mitochondria and chloroplast, and the other five members (*MORF1*, *MORF3, MORF4, MORF6,* and *MORF7*) are targeted to mitochondria, *MORF8* can interact respectively with *MORF1* and *MORF2* in mitochondria and chloroplast [14, 22]. The chloroplast-located MORF2 and MORF9 proteins form homo- and heter- dimers and can affect the RNA editing of *NADH dehydrogenase subunit 4* (*ndhD*) in chloroplasts [23, 24]. *OsMORF9* plays a critical role in the biogenesis of chloroplast ribosomes, chloroplast development, and seedling survival [25]. It has been reported that the *PtrMORF* genes responded to drought in poplar [9]. *OsMORF* gene expression was proved to be affected by cold and salt stresses in rice [19]. In *Nicotiana tabacum*, *NbMORF8* was reported to negatively regulate plant immunity to pathogens [21].

Although the stresses such as infections, high salt, low temperature, and draught are major limiting factors for peach production worldwide, the underlying response mechanisms particularly regarding the roles of RNA editing events remain unclear. Accordingly, we studied *MORF* genes as RNA editing factors in the peach genome based on public transcriptome data. In this tudy, we performed a meta-analysis to evaluate the expression pattern of these *MORF* genes and RNA editing profiles under pathogens infection and irradiation stresses. We observed apparent responses of RNA editing extent and *MORF* gene expression to both stresses and identified three candidate upstream transcription factors that may regulate plant immunity by modulating the *MORF* gene expression. The results provide novel insights into the biological functions of *MORF* genes in peaches and will help elucidate the roles of RNA editing in plant immunity.

## Results

### Characteristics and classification of PpMORF genes in peach

**Fig. 1:**
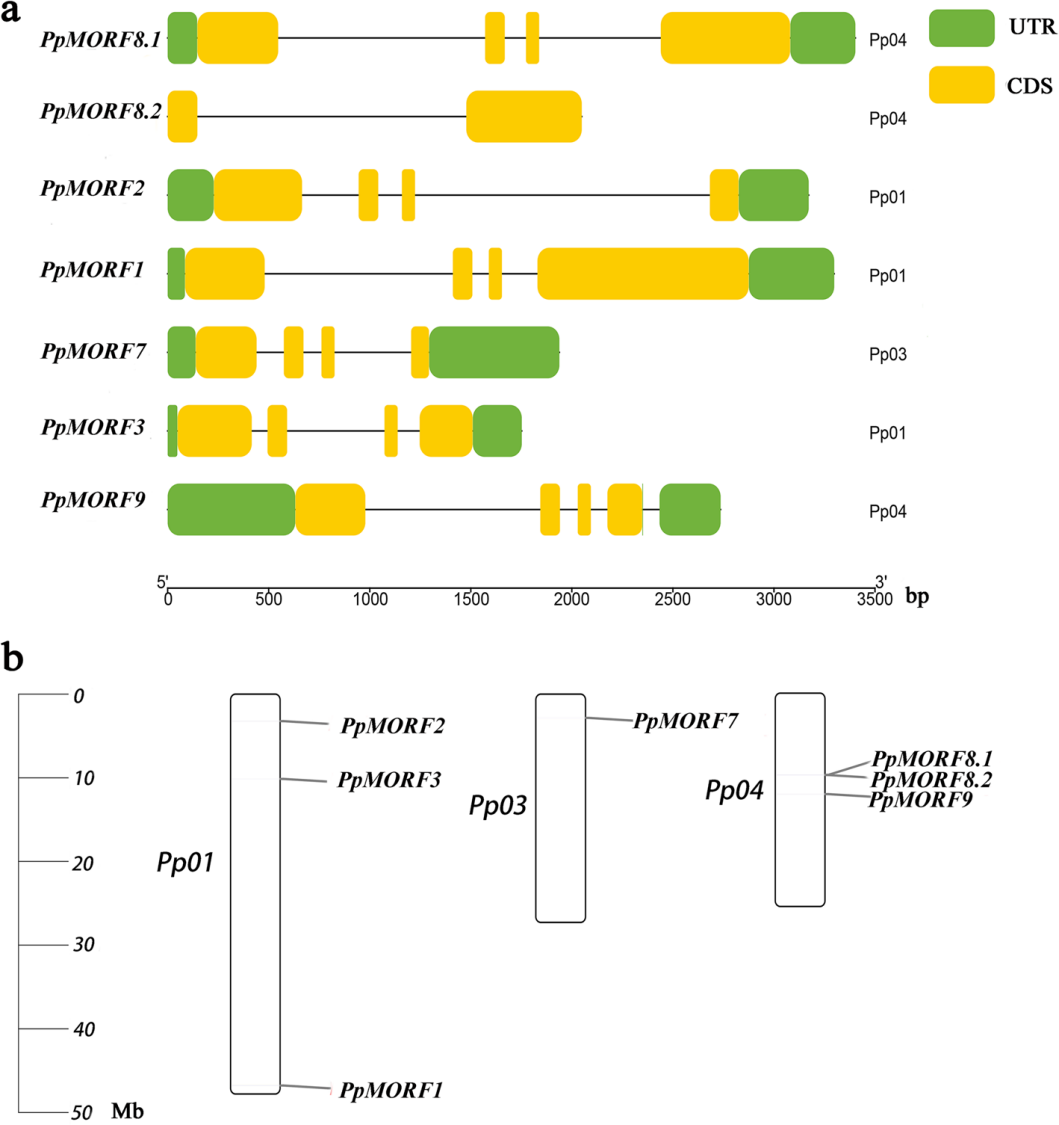
Genomic structures and chromosomal locations of *PpMORF* genes. **a** The genomic structures of *PpMORF* genes. CDSs and UTRs are indicated by yellow and green boxes. The gene sizes are estimated using the length scale at the bottom. **b** The chromosomal locations of the *PpMORF* genes

**Fig. 2:**
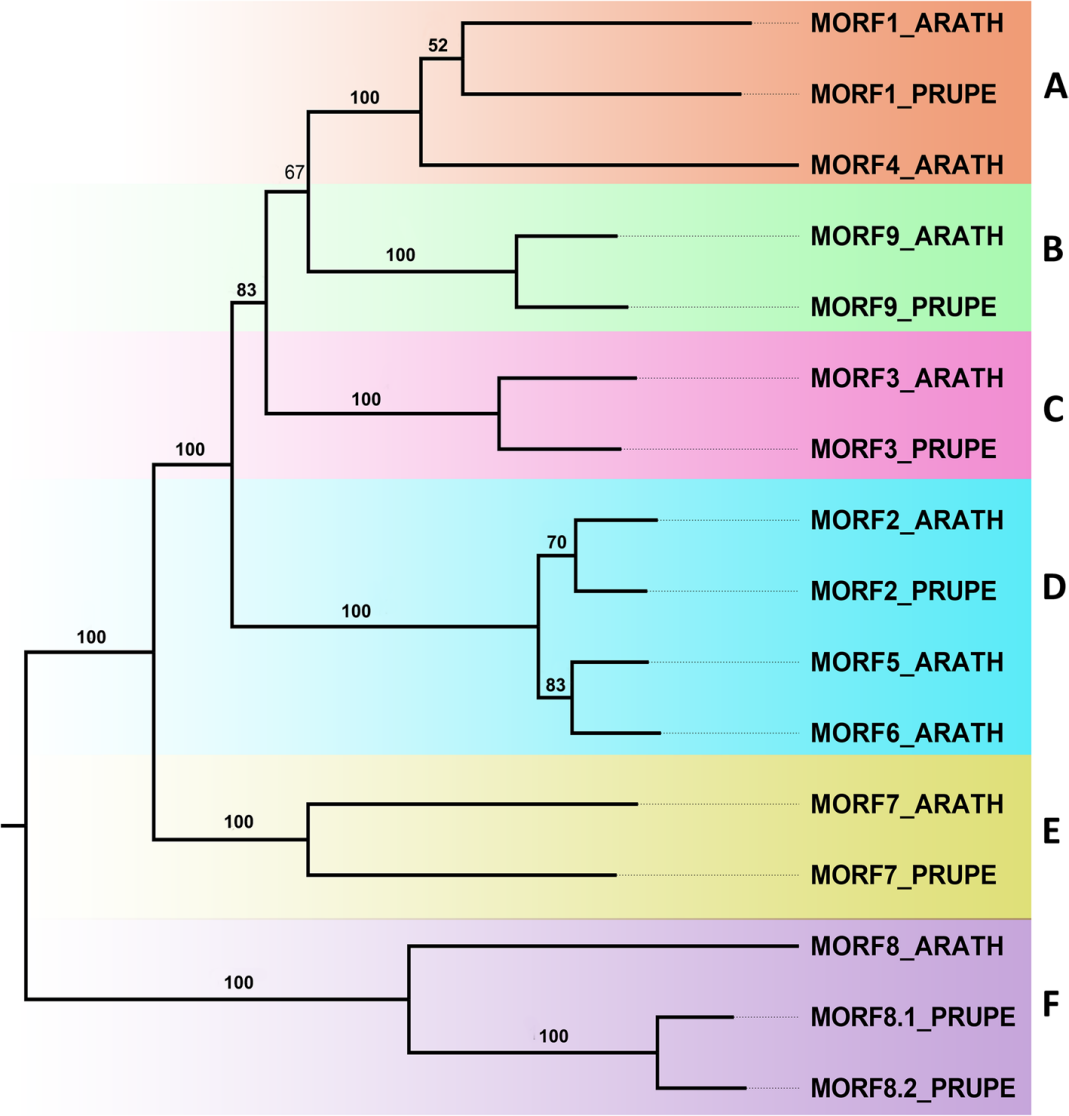
Phylogenetic relationships of the *MORF* gene family from peach and *Arabidopsis*. The full-length amino acid sequences were used for phylogenetic tree construction by the maximum likelihood (ML) method [27]. All the *MORF* genes were classified into six groups and designated as Group **A**-**F**. Branches from different groups are indicated by different colors, the Bootstrap values are indicated on the branches

We searched the peach genome with known *Arabidopsis* MORF proteins as queries, BLASTP and HMM searches [26] were both performed against the entire protein sequences, thus, there were seven *MORF* genes identified in the peach genome (Table 1). All peach *MORF* genes were mapped to the peach reference genome (Fig. 1a**)**, which indicates that these *MORF* genes are distributed in three peach chromosomes, including chromosomes 1, 3 and 4 (Fig. 1b). Based on the full-length amino acid sequences, a phylogenetic tree (Fig. 2) was constructed using the MEGA with maximum likelihood (ML) method [27]. Based on their phylogenetic relationship with *MORF* genes of *Arabidopsis*, we named them *PpMORF1* (Prupe.1G574100), *PpMORF2* (Prupe.1G045300), *PpMORF3* (Prupe.1G130500), *PpMORF7* (Prupe.3G039400), *PpMORF8.1* (Prupe.4G168200), *PpMORF8.2* (Prupe.4G168400) and *PpMORF9* (Prupe.4G197100), accordingly. All the *PpMORF* genes were classified into six groups designated as A-F, nearly all the groups contain only one MORF copy except Group F, which contains two MORF copies including *PpMORF8.1* and *PpMORF8.2.* The exon number of *MORF* genes range is mostly 4, except for *PpMORF8.2*, the encoded protein's length range from 187 to 537 amino acids (Table 1). Subcellular location prediction results showed that *PpMORF2* and *PpMORF9* were localized in the chloroplast, and *PpMORF8.2* is localized in the nucleus, whereas the other five *PpMORF* genes were localized in the mitochondrion (Table 1), they shared the similar subcellular localization with their homologs in *Arabidopsis*.

**Table 1:** The characteristics of putative *PpMORF* genes in peach

Gene Name	Gene ID	Chromosome	Length of protein (aa^a^)	Predicted subcellular location
*PpMORF8.1*	Prupe.4G168200	4	404	mitochondrion
*PpMORF3*	Prupe.1G130500	1	267	mitochondrion
*PpMORF9*	Prupe.4G197100	4	229	chloroplast
*PpMORF1*	Prupe.1G574100	1	537	mitochondrion
*PpMORF2*	Prupe.1G045300	1	250	chloroplast
*PpMORF7*	Prupe.3G039400	3	187	mitochondrion
*PpMORF8.2*	Prupe.4G168400	4	242	nucleus

^a^*aa* Amino acid

### Identification of chloroplast RNA editing sites in peach

Two sets of public RNA-seq data from peaches were used to identify the chloroplast RNA editing sites. One set is about the samples of *Xap* infected leaves from two different peach varieties (‘Jh Hale’ and ‘Redkist’) with accession number SRP108345 [28], and the other set is about the samples of UVB irradiated peach fruits with accession number SRP103523 [29]. We used a combination of Bcftools 'mpileup' and GATK 'HaplotypeCaller' for variant calling, only the SNP sites that quantified the filter criterion of GATK were kept for the detection of RNA editing sites [30, 31]. Following the protocol in our previous study [11], a total of 79 chloroplast RNA editing sites that occurred in 35 genes were detected in peach leaves (Table S1), whereas 42 RNA editing sites that occurred in 26 genes were detected in peach fruits (Table S2). These results indicated the tissue specificity of RNA editing that fewer RNA editing sites in fruits than in leaves. Take the chloroplast RNA editing in leaves as an example, the average editing efficiency is 70.6%, nearly all of the editing types are C-to-U substitutions except for several mismatches, such as *atpA_82* with A-to-C substitution type, which may result from sequencing error. We observed that the RNA editing efficiency varied among individual edited genes, ranging from 10 to 100%, such as the editing efficiency of *rpl16*_4 site is 13.4%, whereas it is nearly 100% for *ndhB_50* site. In addition, several genes have more editing sites, especially *NADH dehydrogenase subunit 2* (*ndhB*) gene and *NADH dehydrogenase subunit 4* (*ndhD*) genes; *ndhB* has 10 editing sites while *ndhD* has 6 editing sites. All the sites of *ndhB* gene (*ndhB*_494, *ndhB*_246, *ndhB*_242, *ndhB*_204, *ndhB*_196, *ndhB*_181, *ndhB*_156, *ndhB*_50, *ndhB*_737, *ndhB*_149) were C-to-U editing type. We observed that the amino acid changes tend to be hydrophobic, such as proline-to-leucine and serine-to-leucine.

### Response of PpMORF gene expression to Xap infection in peach

**Fig. 3:**
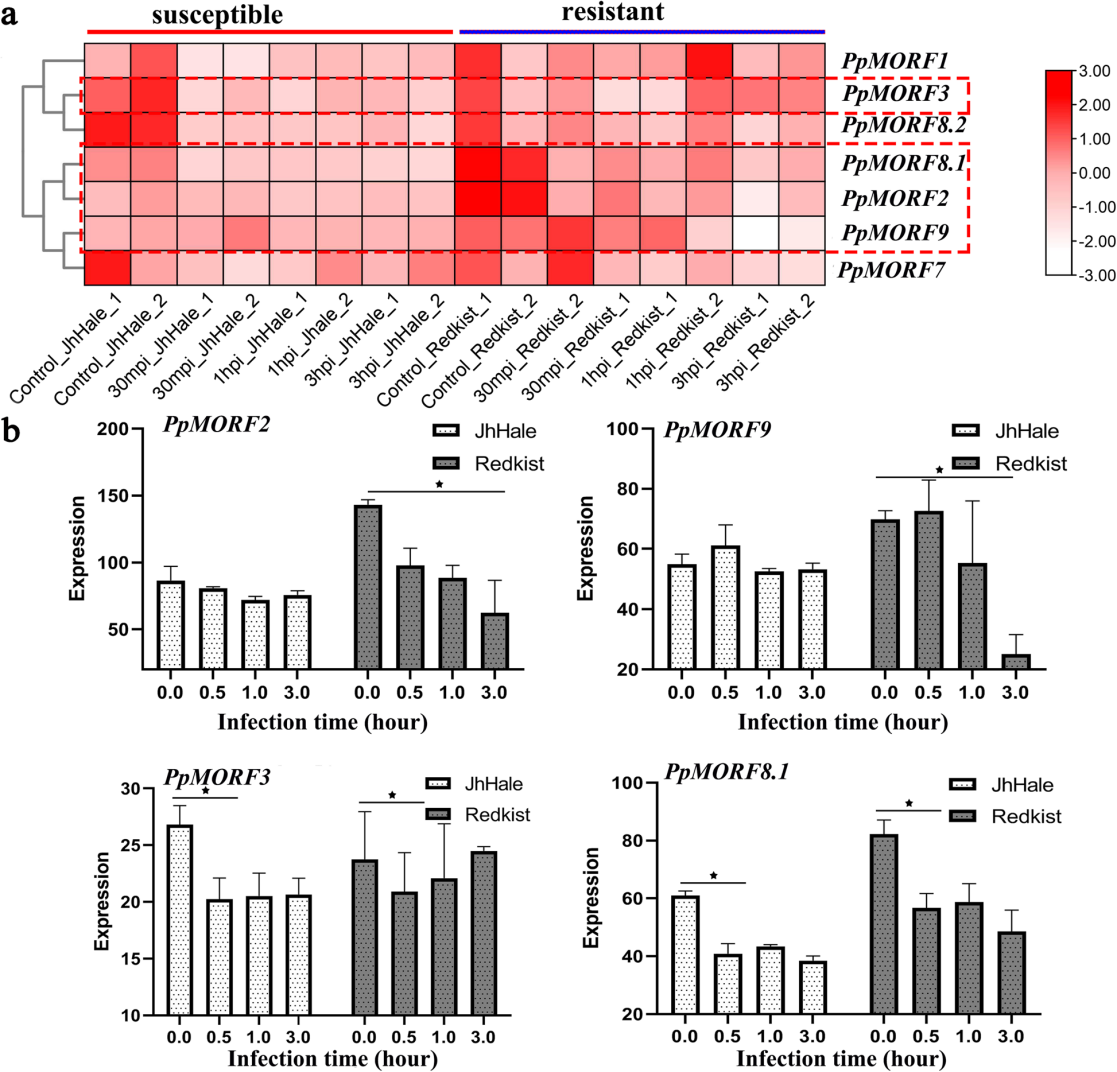
Expression pattern of *PpMORF* genes between resistant (‘Redkist’) and susceptible (‘Jh Hale’) peaches in response to *Xap* infection. **a** Heat map of *PpMORF* gene expression between resistant and susceptible peaches after *Xap* infection. The x-axis represents hours after *Xap* infection (0, 30 min, 1 h, 3 h), and the y-axis represents *PpMORF* genes. The rows were clustered based on expression values. ‘Jh Hale’ and ‘Redkist’ represent susceptible and resistant peach, respectively; (**b**) Expression level of representative *PpMORF* genes (*PpMORF2*, *PpMORF9*, *PpMORF3*, and *PpMORF8.1*) between resistant and susceptible peaches in response to *Xap* infection. Asterisks denote significant differences: *p-value < 0.05

Based on RNA-seq data in resistant (‘Redkist’) and susceptible (‘Jh Hale’) peaches during *Xap* early infection (SRP108345), we examined the response of *PpMORF* genes in expression at four time points. We found that most *PpMORF* genes exhibited reduced expression tendency in both resistant and susceptible peach varieties under *Xap* stress, particularly in resistant peaches (Fig. 3 and Table S3). For ‘Redkist’, at the initial stage after *Xap* infection, most *PpMORF* genes were highly expressed, while the expressions of most *PpMORF* genes were down-regulated at three hours after pathogen infection (Fig. 3a), particularly for *PpMORF9* and *PpMORF2* (Fig. 3b). However, for the susceptible (‘Jh Hale’) peach, the number of down-regulated *MORF* genes was less than that of the resistant peach, although most *PpMORF* genes were down-regulated, but the down-regulation of *PpMORF9* and *PpMORF2* genes was insignificant. Whereas for other *PpMORF* genes, including *PpMORF3, PpMORF8.1, PpMORF8.2, PpMORF1* and *PpMORF7*, they were generally down-regulated in both resistant and susceptible peach varieties, even at the initial stage of *Xap* infection (30 min). These results indicated that most *PpMORF* genes demonstrated down-regulated expression in response to *Xap* infection, especially in resistant peaches, and it’s speculated the changed expression of *PpMORF* genes improved the ability to regulate stress response. From the heatmap plotting (Fig. 3a**)**, we also observed that *PpMORF2* and *PpMORF9* shared similar tissue expression patterns, indicating that *PpMORF9*, and *PpMORF2* genes may work together and only play key roles in resistant peach variety. Interestingly, *PpMORF9 and PpMORF2* only showed reduced expression in resistant peaches are both located in chloroplast, whereas the other *PpMORF* genes (*PpMORF3*, *PpMORF8.1*, *PpMORF8.2*, *PpMORF1*, and *PpMORF7*) that showed reduced expression in both peach varieties are localized in the mitochondrion, indicating the *PpMORF* genes in chloroplast rather than mitochondrion play a more important role in stress response and resistance.

### Response of RNA editing to Xap infection in peach

**Fig. 4:**
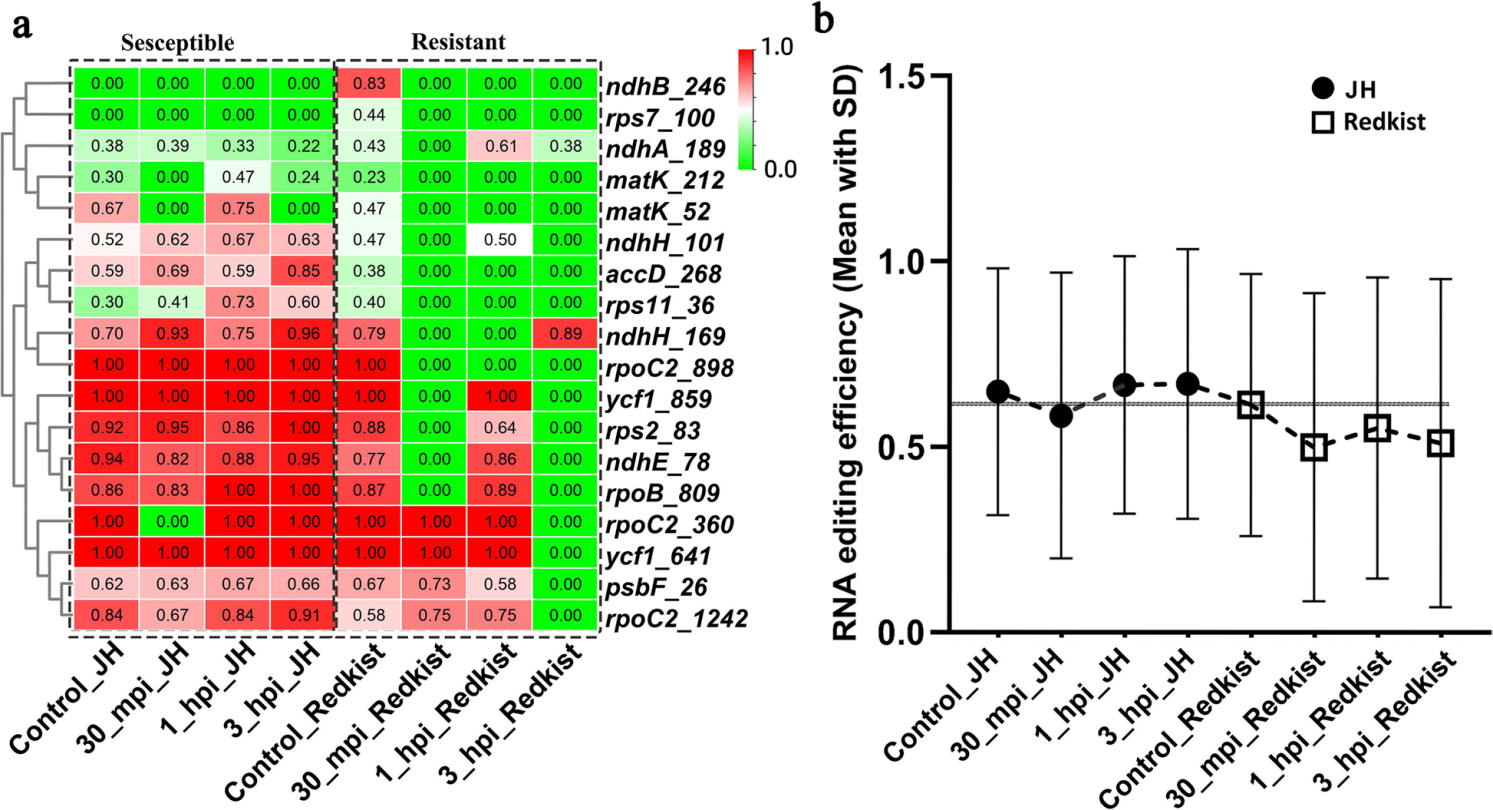
**a** Heat map of RNA editing efficiency in peach chloroplast genes between resistant (‘Redkist’) and susceptible (‘Jh Hale’) peaches in response to *Xap* infection. The x-axis represents infection time points, the y-axis represents chloroplast RNA editing sites, and the rows were clustered based on RNA editing efficiency. **b** RNA editing efficiency in peach chloroplast genes between resistant and susceptible peaches in response to *Xap* infection

Considering the different expression patterns of *PpMORF2* and *PpMORF9* between two peach varieties in response to *Xap* infection (SRP108345), we further analyzed the corresponding chloroplast RNA editing events based on transcripts’ variants. As shown in Fig. 4a, ‘Jh Hale’ and ‘Redkist’ shared a comparable RNA editing pattern at 0 h, while with the *Xap* infection, RNA editing in ‘Redkist’ exhibited a more prominent reduction than that of ‘Jh Hale’. In ‘Redkist’, RNA editing in sites such as *ndhB_246*, *ndhH_169*, *rpoC2_898*, *ycf1_859*, *rps2_83*, *ndhE_78*, *and rpoB_809* were completely lost at 30 min after infection, while editing in sites such as *rpoC2_360*, *ycf1_641*, *psbF_26*, and *rpoC2_1242* were completely lost at 1 h after infection. However, these notable losses of RNA editing were not detected in ‘JH Hale’. In addition, the RNA editing level of all sites showed that the average RNA editing frequency of ‘Redkist’ was slightly lower than that of ‘Jh Hale’ (Fig. 4b**)**. At 0 h, the average RNA editing frequency of ‘JH Hale’ was 0.68, and that of ‘Redkist’ was 0.61. After 30 min, the mean RNA editing frequency of both ‘JH Hale’ and ‘Redkist’ decreased, but the decrease of ‘Redkist’ was significantly greater. The RNA editing frequency of ‘Redkist’ dropped 18% from 0.61 to 0.50, and ‘JH Hale’ dropped 12% from 0.68 to 0.60. At 3 h after infection, the mean RNA editing frequency of ‘Redkist’ was significantly lower than the initial value (Decreased by 14.9%) with ~ 0.5, while the mean RNA editing frequency of ‘JH Hale’ was slightly higher than the initial value (increase by 0.8%). In addition, to rule out the influence of RNA-seq data abundance on the difference in RNA editing events, we also measured and compared gene expression levels of RNA editing genes. However, for RNA editing genes, no expression difference was detected under different treatments, suggesting that stress only affects the RNA editing events and has no influence on the expression level for those genes. Hence, compared with 'JH Hale', the resistant peach variety ‘Redkist’ demonstrated a sharper response to *Xap* infection in RNA editing levels than ‘JH Hale’, which is consistent with their different expression level of *PpMORF* genes in the chloroplast. Down-regulation of *PpMORF2* and *PpMORF9* may be in charge of the reduced chloroplast editing in ‘Redkist’. Under pathogen infection, the chloroplast *PpMORF* genes were prone to be down-regulated, thereby reducing the RNA editing level to trigger a series of defense responses and increase the resistance.

### Response of PpMORF gene expression and RNA editing to UVB irradiation in peach

**Fig. 5:**
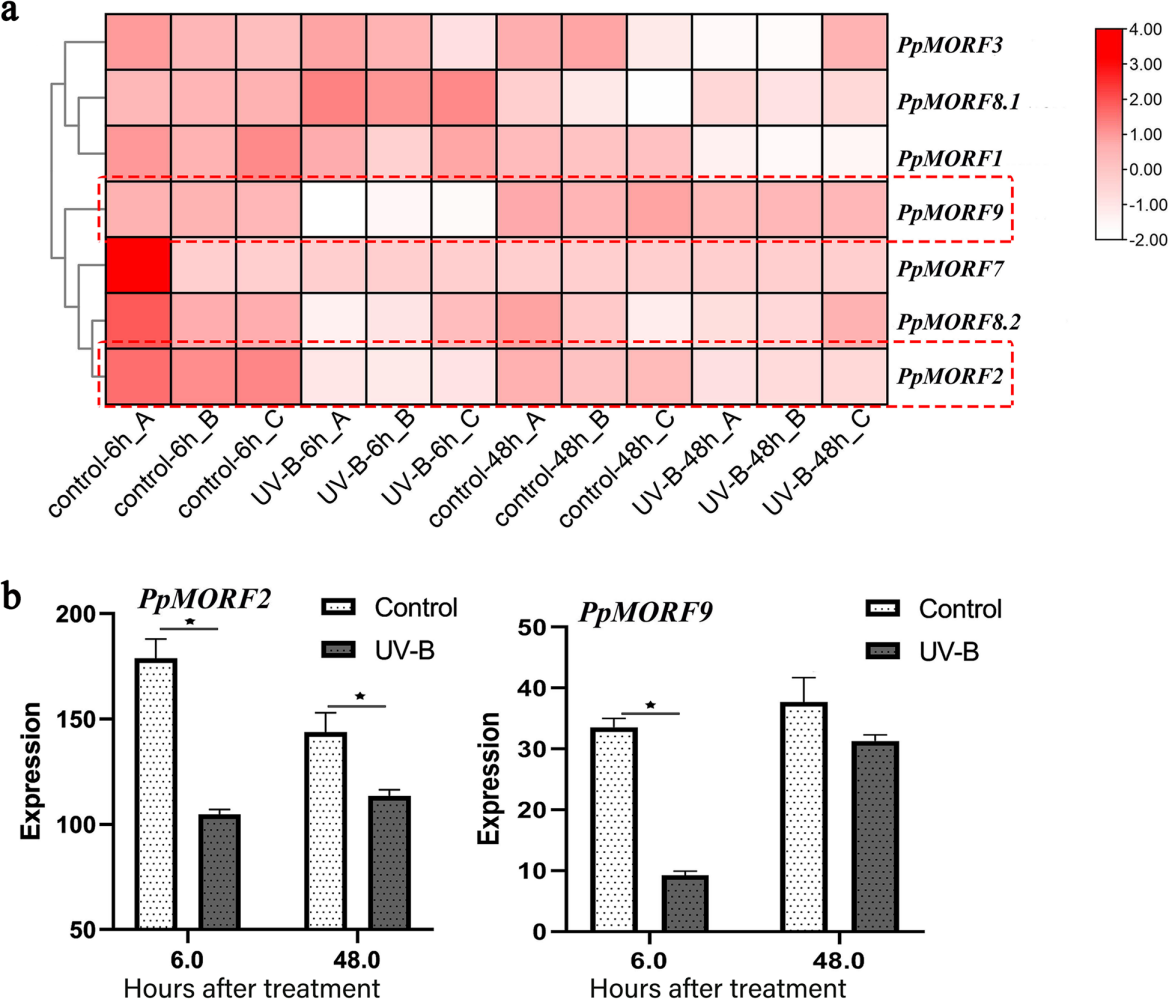
Expression patterns of *PpMORF* genes in peach fruits after UVB irradiation. **a** Heat map of *PpMORF* gene expression in peach fruits after UVB irradiation. The x-axis represents hours after UVB irradiation (6 h, 48 h), and the y-axis represents *PpMORF* genes. The rows were clustered based on expression values. **b** The expression level of *PpMORF* genes (*PpMORF2* and *PpMORF9*) in peach fruits after UVB irradiation. Asterisks denote significant differences: *p-value < 0.05

We further examined the response of *PpMORF* gene expression under abiotic stress based on RNA-seq data in samples of UVB-irradiated peach fruits (SRP103523). In comparison with the control group, several *PpMORF* genes exhibited significantly reduced expression levels in UVB-irradiated peaches, especially for *PpMORF2* and *PpMORF9* (Fig. 5a and Table S3). At 6 h after UVB irradiation, the expression values of *PpMORF2* and *PpMORF9* decreased significantly, whereas, at 48 h after UVB irradiation, the expression values of *PpMORF2* and *PpMORF9* in UVB-irradiated peaches increased slightly compared with that of 6 h, but was still lower than those in the control peaches (Fig. 5b). The above observation indicated that peach *PpMORF* genes also exhibited down-regulated expression under UVB irradiation stress, especially for those chloroplast *PpMORF* genes, suggesting their roles in abiotic stress responses.

**Fig. 6:**
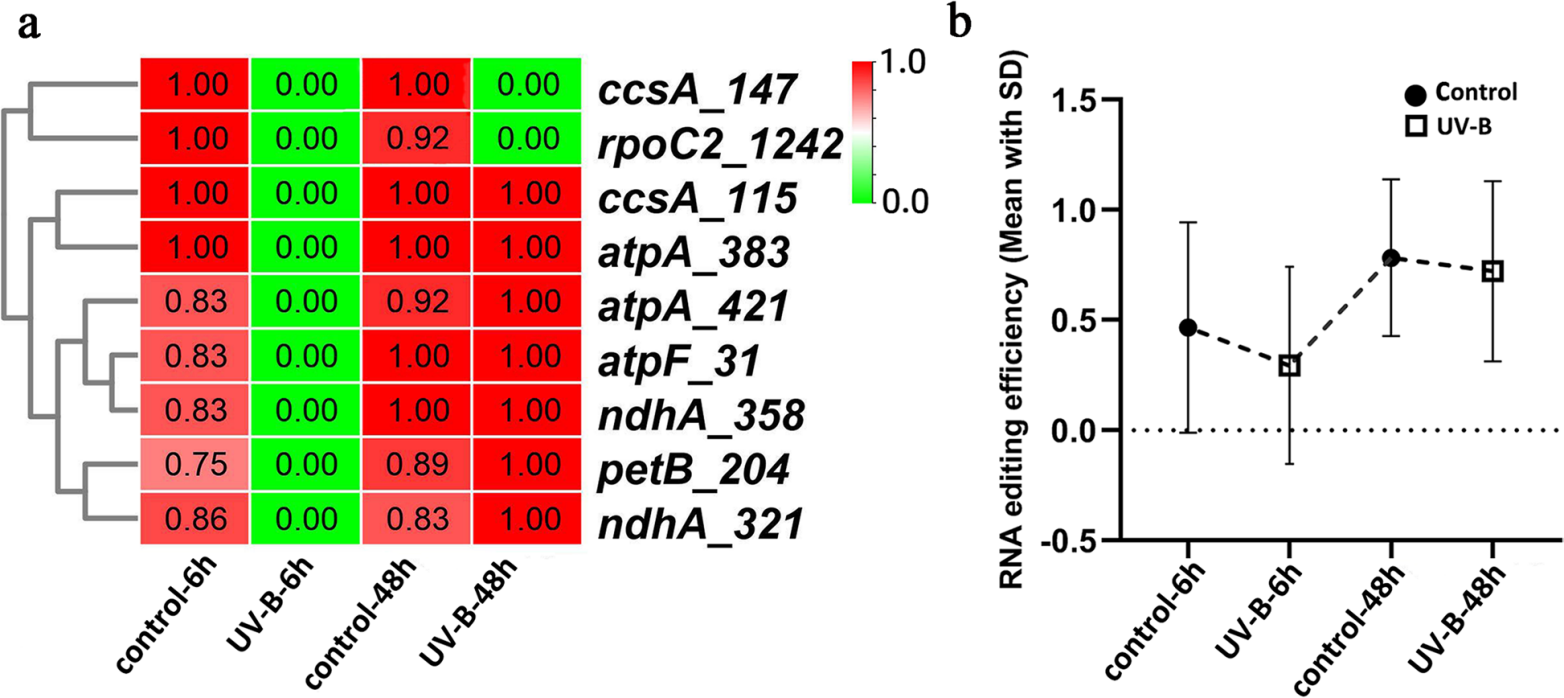
Response of chloroplast RNA editing to UVB irradiation in peach fruits. **a** Heat map of RNA editing efficiency in peach chloroplast genes after UVB irradiation. The x-axis represents time points after UVB irradiation, the y-axis represents chloroplast RNA editing sites, and the rows were clustered based on RNA editing efficiency. **b** Comparison of RNA editing efficiency in peach chloroplast genes after UVB irradiation

Considering the reduced expression of *PpMORF2* and *PpMORF9* under UVB irradiation stress, we further analyzed the corresponding chloroplast RNA editing events (Fig. 6a). At 6 h after UVB irradiation, we observed that UVB-irradiated peaches exhibited a wide loss of editing sites, editing in sites *ccsA_147*, *rpoC2_1242*, *ccsA_115*, *atpA_383*, *aptA_421*, *atpF_31*, *ndhA_358*, *petB_204*, *ndhA_321* were completely lost in comparison with the control group. While at 48 h after UVB irradiation, most of the lost RNA editing such as *ccsA_115*, *atpA_383*, *aptA_421*, *atpF_31*, *ndhA_358*, *petB_204*, and *ndhA_321* returned to normal levels in the UVB-irradiated peaches. However, there were still several sites without editing including sites of *ccsA_147*, *rpoC2_1242*. A comparison of RNA editing frequency was further conducted. At 6 h after UVB radiation, the average RNA editing frequency in UVB-irradiated peaches was 33.3% lower than that of control peaches (Fig. 6b), while at 48 h after UVB radiation, the RNA editing frequency in UVB-irradiated peaches increased slightly, but was still 7.6% lowered than that of the control group. These observations revealed that chloroplast RNA editing exhibitted a reduction tendency under UVB irradiation stress, which is also consistent with the reduced expression level of chloroplast *PpMORF* genes. At the initial stage, the UVB irradiation stress elicits down-regulation of chloroplast *PpMORF2* and *PpMORF9*, which further affect the normal RNA editing, the reduced RNA editing level may trigger a series of defense responses to stress. After some time, with the stress relieves, both the expression of *PpMORF* genes and RNA editing rebound to the normal state. Therefore, as the key elements of RNA editing, *PpMORF* genes may modulate the editing level and functions of chloroplast genes, thus providing a flexible strategy to increase stress tolerance.

### Upstream transcription factors associated with PpMORF genes in peach

**Fig. 7:**
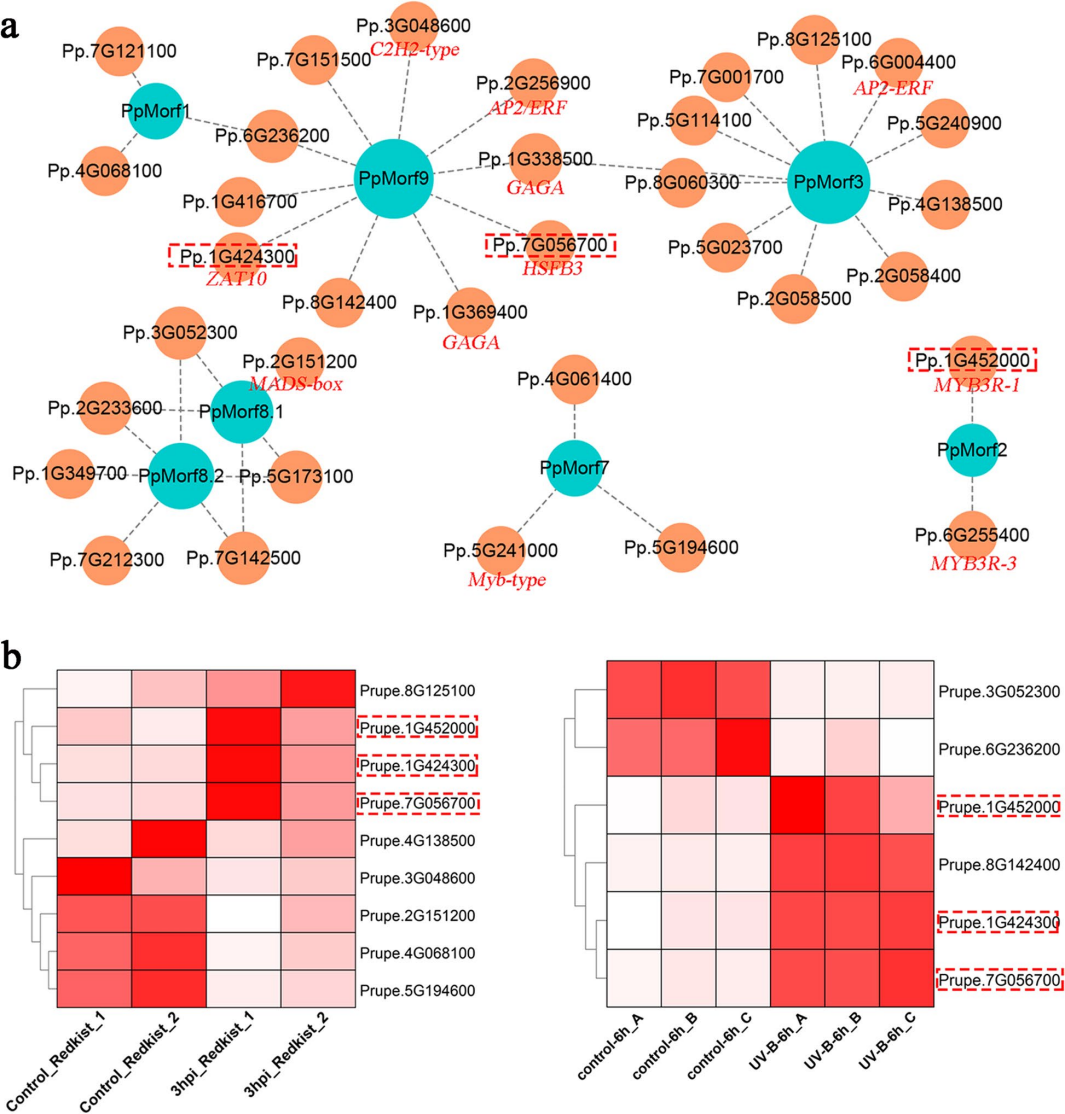
Upstream transcription factors associated with *PpMORF* genes in peach. **a** The regulatory network between *PpMORF* genes and transcription factors. The nodes of *PpMORF* genes and transcription factors are denoted by blue and red circles, respectively. The below red fonts represent the gene names of transcription factors. **b** Expression patterns of *PpMORF* genes’ upstream transcription factors in response to stress in peach. The left panel shows the response of expression to *Xap* infection (3 h after *Xap* infection in ‘Redkist’), while the right panel shows the response of expression to UVB irradiation (6 h after UVB irradiation). The transcription factors shared by both conditions are marked by the red dashed line

To investigate the underlying pathway that may regulate the *PpMORF* gene expression, we obtained 34 upstream transcription factors of *PpMORF* genes from the PlantRegMap database [32–34]. The regulatory interaction between transcription factors and *PpMORF* genes showed that the *PpMORF3* gene had the most transcription factors with 11, followed by *PpMORF9* with 10, *PpMORF2* gene has the fewest transcription factors with only two (Fig. 7a**)**. *PpMORF9* shares one transcription factor with *PpMORF1* and *PpMORF3* respectively, and two copies of the *PpMORF8* gene share four transcription factors. Based on two types of RNA-seq data under different treatments (biotic and abiotic stresses), differential expression analysis was conducted against these transcription factors, and a total of nine and six transcription factors showed differential expression under biotic (3 h after *Xap* infection for ‘Redkist’) and abiotic (6 h after UVB irradiation) stresses, respectively (Fig. 7b). There were three common transcription factors up-regulated in both conditions, including the *myb 3R-1 gene* (*MYB3R-1*, Pp.1G45000), *zinc finger gene* (*ZAT10*, Pp.1G424300), and *heat stress transcription factor B-3* (*HSFB3*, Pp.7G056700). Interestingly, these three transcription factors only negatively regulate the expression of *PpMORF2* and *PpMORF9*. These observations suggested that those differentially expressed transcription factors, especially the shared ones, may participate in the upstream regulation of chloroplast *PpMORF* gene expression and RNA editing in response to both biotic and abiotic stresses.

## Discussion

Generally, plant RNA editing appears to act as an indirect repair mechanism to correct DNA mutations on the RNA level by restoration of conserved amino acids to guarantee proper protein function [35]. Previous studies showed plant RNA editing played multiple roles during plant developmental processes [36], organelle biogenesis [37], plant flowering [38], response to particular environmental conditions [39] and signal transduction [40]. However, the underlying mechanism still needs further clarification. MORF proteins have been identified as essential components of plant RNA editosome through interacting with other RNA editing factors, PPR proteins, organelle RNA recognition motif (ORRM) proteins, organelle zinc-finger (OZ) proteins, and protoporphyrinogen oxidase 1 (PPO1) [8, 14]. Recent evidence suggested that *MORF* genes played a critical role in plant development and stress response, such as seedling survival in rice, drought stress in poplar, and pathogen stress in tobacco and kiwifruit [9, 13, 19, 21]. It’s determined that *NbMORF8* localized in mitochondrion negatively regulates plant immunity to *Phytophthora* pathogens [21] and indicated that nuclear gene regulation in plant enhanced resistance. Hence, the roles of RNA editing in response to stresses may be partly explained from the perspective of the function of MORFs.

Utilizing the recently released peach assembly, a total of seven members of *PpMORF* genes were identified in this study. Similar to *Arabidopsis*, *PpMORF* genes in peaches mostly were localized in mitochondria and a few in chloroplasts. *PpMORF2* and *PpMORF9* are exclusively localized in chloroplast, whereas *PpMORF1*, *PpMORF7*, *PpMORF3*, and *PpMORF8.1* are localized in mitochondria, however, as a duplicated copy, *PpMORF8.2* changed to be located in the nucleus. The similar expression patterns between *PpMORF2* and *PpMORF9* further confirmed their functional relevance and selective heteromer interactions. Based on two types of public RNA-seq data under different treatments (biotic and abiotic stresses), we conducted a meta-analysis to examine the roles of RNA editing in plant immunity, obvious response of *PpMORF* genes and varied chloroplast RNA editing profiles were observed, especially the reduced expression of *PpMORF2* and *PpMORF9*. Their varied response to pathogens infection was also detected between resistant and susceptible peach cultivars, indicating the roles of *PpMORF* genes as controlling elements in stress response by modulating the chloroplasts RNA editing extent in plant immunity. The varied disease resistance capacity between resistant and susceptible peaches partly can be explained by their discrepancy of *MORF* gene expression in response to pathogen infection. In addition, for RNA editing genes in chloroplasts, no expression difference was detected under different treatments, suggesting that stress only affects the RNA editing events and has no influence on the expression level for those genes. Hence, it is reasoned that the response of RNA editing events may be regulated by the expression of RNA editing factors. Several transcription factors that regulate the expression of peach *MORFs* were also identified in our study, such as *ZAT10*, *MYB3R-1*, *HSFB3,* and so on. *ZAT10* is a transcriptional repressor involved in abiotic stress responses, a previous study confirmed plants overexpressing *ZAT10* showed growth retardation and enhanced tolerance to drought, salt, heat, and osmotic stresses [41]. *MYB3R-1* is a transcription repressor that regulates organ growth, it specifically binds DNA sequence 5'-AGAAnnTTCT-3' known as heat shock promoter elements [42]. Those differentially expressed transcription factors may participate in the upstream regulation of chloroplast *MORF* gene expression and RNA editing in response to both biotic and abiotic stresses.

**Fig. 8:**
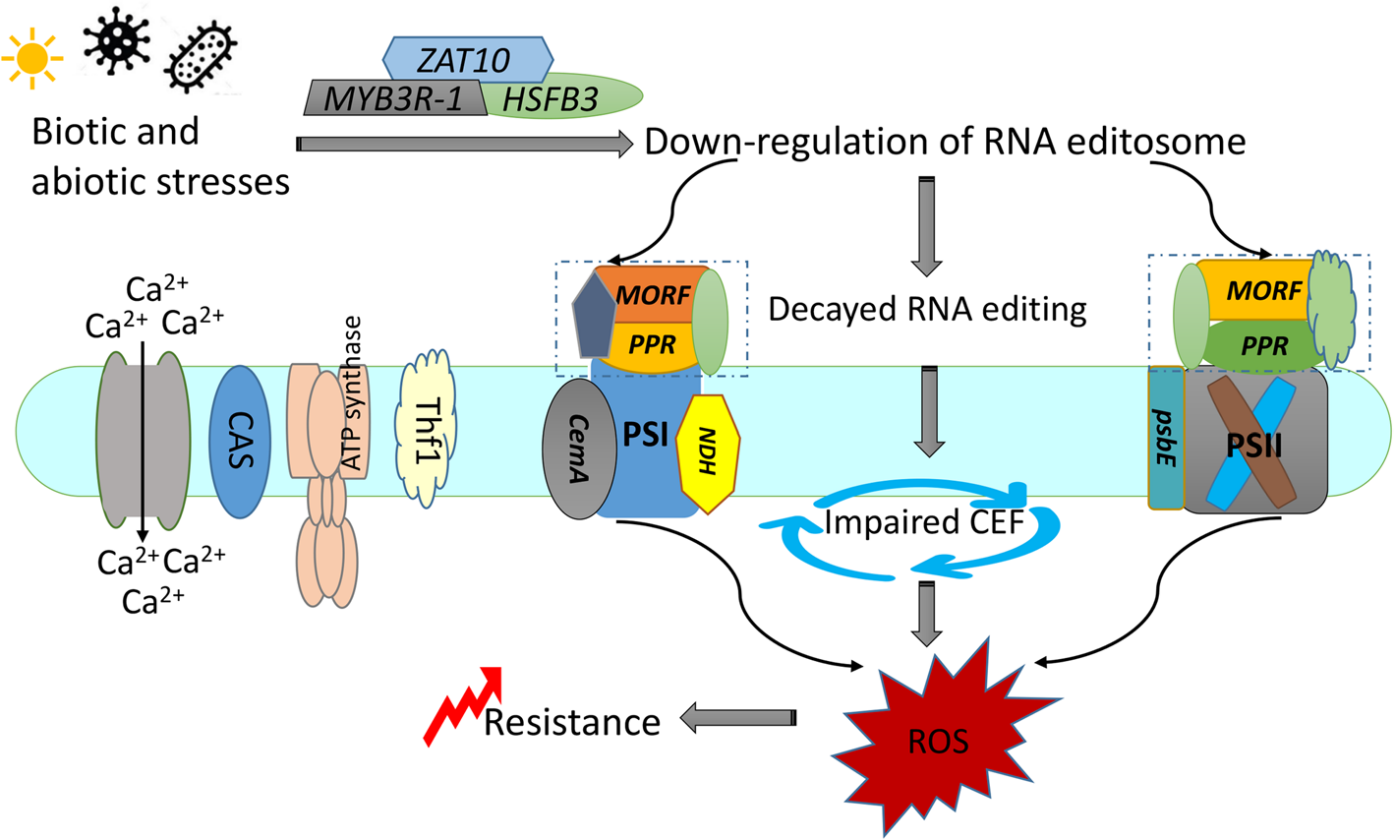
Schematic model for the role of *MORF* genes in plant immunity. The MORF-regulated ROS burst is likely achieved through its effect on the functionality of Photosystem I and II. Upstream transcription factors regulate the expression of *MORF* genes. *MORF* genes participate in the RNA editing of chloroplast photosystem genes and subsequently affect the cyclic electron flow activities. Stress such as pathogen infection and UVB irradiation leads to the down-regulation of *MORF* genes and reduced RNA editing efficiency, thereby impairing CEF, and up-regulating ROS levels, Calcium influx, which enhances the immunity to stress

Chloroplasts play key roles in plant-pathogen interactions and are important for reactive oxygen species (ROS) that act as key defense molecules in plant immune responses [43]. However, it remains largely unclear how chloroplast proteins achieve modulation of the plant immune system. Recently, nuclear gene expression has been acknowledged to be involved in the post-transcriptional regulation of chloroplast function in response to external stimuli, and RNA editing is one such control mechanism [39]. It’s confirmed that *overexpressor of cationic peroxidase3* (*ocp3)* which is targeted to chloroplasts contributes to control over the extent of *ndhB* transcripts editing and proposed that *ocp3* mediated chloroplast RNA editing in plant immunity. *ndhB* encodes the B subunit of the chloroplast NADH dehydrogenase-like complex (NDH) involved in cyclic electron flow (CEF) around photosystem I. *Ocp3-silenced* mutants lead to *ndhB* editing efficiency decays, thereby impairing CEF and enhancing disease resistance to pathogens substantially [39]. In our study, the affected chloroplast genes with reduced RNA editing in response to stresses mostly function in DNA-RNA transcription and RNA splicing and photosystem, such as *ndhB*, *ndhH, ndhD*, *rpoC*, *ndhE*, and *rpoB. ndhB* encodes the B subunit of the chloroplast NADH dehydrogenase-like complex (NDH) involved in CEF around photosystem I. NDH complex activity and plant immunity appear as interlinked processes. *MORF* genes, similar to *ocp3*, may modulate the plant-pathogen interaction by controlling the extent of chloroplast RNA editing, especially components of the NDH complex. Hence, we speculated the decays of editing efficiency in these genes might trigger the impaired CEF, thereby leading to the activation of ROS-mediated retrograde signaling, and the disease resistance to pathogens or other stresses substantially enhanced (Fig. 8). However, we also found that this regulatory strategy is flexible from the result of response to UVB irradiation. If the stress relieves, both the expression of *MORF* genes and RNA editing return to the normal state. There is a ‘trade-off’ between resistance and homeostasis.

## Conclusions

The present study is a comprehensive meta-analysis of the *PpMORF* gene family in peaches, particularly for their roles in response to biotic and abiotic stresses. We identified seven *PpMORF* genes in total and performed a series of analyses of their basic structures, classification, chromosomal, subcellular localization, and expression. The findings revealed that most *PpMORF* genes were localized in mitochondria or chloroplast, with one in the nucleus. In response to different stresses, including pathogen infection and UVB radiation, chloroplast localized *PpMORF* genes exhibited down-regulated expression, accompanied by reduced chloroplast RNA editing. In addition, different expressions of *MORF* genes and RNA editing profiles in chloroplasts between resistant and susceptible peaches after pathogen infection were also observed, indicating the contributions of *PpMORF* genes to the disease resistance of different peach varieties. Finally, some transcription factors that regulated *PpMORF* gene expression were predicted, they may play an essential role in the *MORFs-*mediated stress adaption pathway. This study will be highly useful for further molecular elucidation of plant immunity and the breeding of resistant peaches.

## Methods

### Genome-wide identification of PpMORF genes in peach

The peach (*Prunus persica*) genome and annotation files were downloaded from Genome Database for Rosaceae (GDR) (https://www.Rosaceae.org/). We used two searching strategies to obtain peach *MORF* genes. First, using the previously identified *MORF* genes in *Arabidopsis* as queries [14], we implemented BLASTP searches against the entire protein database of peaches with an E-value cut-off of 0.00001 to reduce false positives. Second, Hidden Markove Model (HMM) profiles of *MORF* genes in *Arabidopsis* were constructed and used to search against the peach protein database by using HMMER software [26], nine *MORF* genes in *Arabidopsis* were aligned and used to build HMM profiles using ‘‘hmmbuild’’ command, thus the resulting HMM profiles were used to search against peach protein sequences with an E-value cut-off of 0.001 using ‘hmmsearch’ command. Finally, all the candidate peach *MORF* genes were named based on their phylogenetic relationship with that of *Arabidopsis* accordingly. The phylogenetic tree from full-length amino acid sequences was constructed using the MEGA with maximum likelihood (ML) method [27].

### Gene structure analysis, subcellular and physical localization

TargetP [44] and LOCALIZER (http://localizer.csiro.au/) were used for predicting the putative subcellular localization of peach *MORF* genes. The gene structure and positional information of peach *MORF* genes on the genome were obtained from the annotation documents, and we utilized TBtools [45] to draw the sketch map of gene structure and physical location.

### Transcriptome data collection and preprocessing

Two types of transcriptome data of peaches under different stresses (biotic and abiotic stresses) were downloaded from the Short Read Archive (SRA) database of the National Center for Biotechnology Information (NCBI). The first transcriptome data was collected from resistant ‘Redkist’ and susceptible ‘Jh Hale’ peach leaf samples in response to *Xap* during early infection with accession number SRP108345 [28]. Both cultivars are yellow melting flesh peaches, ‘Redkist’ was obtained from a mutation of ‘Redskin’ and is highly resistant to bacterial spot; ‘JH Hale’ was obtained from self-pollination of ‘Elberta’ and moderately susceptible to *Xap* [28]. The early infection consists of 30 min, 1 and 3 h-post-infection (hpi) after inoculation with *Xap*, and each condition consists of two replicates. The second data is peach fruit samples under different treatments (control; 6 h, 48 h after UVB irradiation) with accession number SRP103523 [29], melting flesh peach (*Prunus persica* L. Batsch cv. Hujingmilu) fruits were harvested in Ningbo, China. Eeach treatment consists of three biological replicates, an average of ~ 11 million clean reads (Q30 > 94.87%) were obtained per sample, with ~ 155 million clean reads in a total of 12 samples with 91.92% mapped to the peach genome. Before analysis of RNA-Seq data, we utilized the FastQC tool to check the quality of the transcriptome data first [46], and trimmed the adapter and low-quality bases (phred score < 33) with Trimmomatic (v0.39) [47], only reads > 40 bp were kept.

### Expression analysis of PpMORF genes in peach in response to stress

The clean reads of RNA-seq data from each sample were mapped against the peach genome reference with HISAT2 [48], and each SAM file was converted into a BAM file and sorted with SAMtools [49, 50]. Further transcript assembly and quantification of the read alignments were performed with Stringtie [51]. Gene expression levels were measured by FPKM (fragments per kilobase of transcript per million mapped reads). The differential expressed genes were determined by using EdgeR [52]. Cluster analysis was also performed using the HeatMap function implemented in TBtools [45] based on the matrix of *MORF* gene expression, which was initially normalized by subtracting the row-wise mean from the values in each row of data and divided by the standard deviation value of each row.

### Identification of RNA editing sites

For RNA editing site detection, we retrieved the genome sequences (NC_014697.1) of peach chloroplast as well as their annotation files from the nucleotide database of NCBI. The transcriptome data were mapped to chloroplast genome reference by using HISAT2 software with default parameters [48]. Afterward, each SAM file was converted into a BAM file, sorted with SAMtools [49, 50]. We used a combination of Bcftools 'mpileup' and GATK 'HaplotypeCaller' for variant calling. Firstly, The variant calling process was conducted by SAMtools ‘mpileup' command, and the single nucleotide polymorphisms (SNPs) were identified by BCFtools ‘call’ command [31]. Secondly, to validate the SNPs, we also employed Genome Analysis Toolkit (GATK, v4.0) to detect SNPs [30], we mapped the clean reads to the reference using BWA-MEM with default parameters [53]; the multiple tools (‘MarkDuplicates’, ‘HaplotypeCaller’ and ‘VariantFiltration’, etc.,) implemented in GATK [30] were used to obtain high-quality SNPs, with strict filter settings “QD < 2.0 || MQ < 40.0 || FS > 60.0 || SOR > 3.0 || MQRankSum < -12.5 || 218 ReadPosRankSum < -8.0”. For chloroplast, based on their SNP-calling results and gene annotation files, RNA editing sites were identified by using the REDO tool [54]. A series of comprehensive rule-dependent and statistical filters implemented in the REDO tool were used to reduce the false positives. Afterward, we further minimize false-positive sites by manually examining all mismatches. To rule out the influence of RNA-seq data abundance on the difference in RNA editing events, we excluded sites with no RNA-seq data in a certain sample. For each site, RNA editing efficiency was quantified by the proportion of edited transcripts in total covered transcripts. The matrix of RNA editing efficiency was initially normalized by subtracting the row-wise mean from the values in each row of data and divided by the standard deviation value of each row, for comparison between conditions, cluster analysis was subsequently performed by using the HeatMap function implemented in TBtools [45].

### Identification of upstream regulatory transcription factors of PpMORF genes

The transcriptional regulatory map of peach was retrieved from the PlantRegMap database [32–34]. Transcriptional regulations in PlantRegMap were identified from the literature and ChIP-seq data, or inferred by combining transcript factors (TF) binding motifs and regulatory elements data, this tool was used to infer potential regulatory interactions between TF and input genes, and found the TFs which possess over-represented targets in the input gene set. We submitted the gene symbols of *PpMORF* genes in the website tools (http://plantregmap.gao-lab.org/network.php) to retrieve corresponding regulations and upstream regulatory transcription factors under default settings. Finally, the regulatory transcription factors were annotated, and the regulatory interactions were further mapped using Cytoscape [55].

## Supplementary Information

**Additional file 1: Table S1.** Detailed information of chloroplast RNA editing sites identified in peach leaves.

**Additional file 2: Table S2.** Detailed information of chloroplast RNA editing sites identified in peach fruits.

**Additional file 3: Table S3.** Detailed information of differential expressed PpMORF genes under Xap infection and UVB irradiation treatments.

## Data Availability

The datasets analyzed during the current study are available in the NCBI repository (https://www.ncbi.nlm.nih.gov/) with accession SRP103523 and SRP108345.

## Abbreviations

C-to-U: Cytosine-to-uracil
PPR: Pentatrico peptide repeat
ndhB: Subunit of the NADH dehydrogenase-like complex
MORF: Multiple organelle RNA editing factors
atpA: ATP synthase alpha-subunit
Xap: Xanthomonas arboricola pv. Pruni
MYB3R-1: Myb 3R-1 gene
ZAT10: Zinc finger gene
HSFB3: Heat stress transcription factor B-3
CEF: Cyclic electron flow
ROS: Reactive oxygen species
TF: Transcript factors
